# Glances at the Hospitals

**Published:** 1902-02-08

**Authors:** 


### GLANCES AT THE HOSPITALS.

### DORSET COUNTY HOSPITAL.

At the beginning of the year the financial accounts showed a balance of £147 against the hospital. The receipts for the year amounted to £2,246, and the expenditure to £2,117, showing a balance of £129 in favour of the year's working, and being almost sufficient to wipe off the adverse balance of the previous year. On January 1st the hospital contained 28 patients, and 283 were admitted during the year, making a total of 311 under treatment. Of these 103 were cured, 92 relieved, 45 were made out-patients 18 were discharged for other reasons, 22 died, and 31 remained in the hospital. The weekly average of resident patients was 27, and the average cost per head per week was 23s. 9d. The medical and surgical tables are provided with columns showing the numbers under treatment for the various diseases, the numbers cured, relieved, incurable, and the deaths. There were 92 surgical operations. Of these cases 59 were discharged recovered, 22 relieved, 1 not relieved, 5 died subsequent to operation, and 5 remained in the hospital. Anæsthetics were administered 138 times. In 27 of these chloroform was the agent, in 37 ether, in 29 a mixture of ether and nitrous oxide, and in 45 nitrous oxide alone.

### TORBAY HOSPITAL AND EYE INFIRMARY.

The income amounted to £1,938, and the expenditure to £2,252. In addition to this deficit of £314 there was an adverse balance of £382 from the previous year. To liquidate this debt the governors issued a special appeal, in answer to which they received a sum of £325. No doubt the balance will soon be forthcoming; but the governors very truly point out that, if the hospital is to continue efficient, more annual subscribers must be obtained, for these are the main reliable source of income. On looking at the sources of income of this hospital we find that the annual subscriptions amounted to £739, the donations for current expenditure to £320, and dividends to £367, but we do not find the workpeople's own contributions set out separately. It is stated the patients paid about £60, and that the Hospital Saturday collections and collection boxes, etc., produced £195. No doubt a considerable proportion of the latter would be contributed by workpeople, but we believe that more might be done in that direction, and when it is seen that the people who chiefly derive benefit from the hospital are increasing their contributions, it is reasonable to suppose that richer people will follow their lead more willingly. During the year there were 389 patients under treatment. Of these 235 were cured, 72 were relieved, 23 were discharged for other reasons, 38 remained in the hospital, and 21 died. The medical and surgical tables show the numbers cured, relieved, died, etc., but it would be desirable in future reports to add an operation table with results stated, and another table showing the anæsthetic used. So in the ophthalmic department. We are told that 80 operations were performed, but no results are stated.

### HASTINGS AND EAST SUSSEX HOSPITAL.

The income was £4,210, but £500 of this sum being a legacy was transferred to the surplus and deficiency account, thus reducing the ordinary income to £3,707. The expenditure was £3,971, which left an adverse balance of £263 on the working account for the year. On January 1st the hospital contained 46 patients, and 635 were admitted during the year, making a total of 681. Of these 428 were discharged cured, 71 discharged relieved, 40 for various reasons, 44 died, and 52 remained on the books. The average cost of each in-patient was £4 7s., the average duration of stay in the hospital was 29 days, the average daily number of beds occupied was 55, and the yearly cost per bed occupied was £54. The total number of out-patients treated during the year was 3,657, and the average cost of each out-patient was 5s. 6d. The medical and surgical tables merely give the numbers under treatment for the various diseases, and might, therefore, almost as well be omitted, as they are useless for purposes of comparison. There were 352 operations performed under anæsthetics, but the agent used is not stated, nor are the results of the operations given. Taking the in- and the out-patients together, a total number of 4,338 was treated during the year.

